# Postoperative complications after central nervous system tumor resection in pediatric patients admitted to an intensive care unit in Colombia

**DOI:** 10.3389/fonc.2024.1491943

**Published:** 2024-12-06

**Authors:** Rubén E. Lasso-Palomino, Inés Elvira Gómez, María José Soto-Aparicio, Andrés Gempeler, Andrés Pombo-Jiménez, Melissa Gómez-Toro, Valentina Rojas-Robledo, María Alejandra Jiménez-Arévalo, Karla Alejandra Bastidas-Toro, Jimena Sierra, Sofía Martínez-Betancur, Camila Ariza-Insignares, Isabella Montaño-Vivas, Ximena Castro, Anita V. Arias

**Affiliations:** ^1^ Fundación Valle del Lili, Unidad de Cuidado Intensivo Pediátrico, Unidad Materno Infantil, Cali, Colombia; ^2^ Universidad Icesi, Facultad de Ciencias de la Salud, Departamento de Pediatría, Cali, Colombia; ^3^ Universidad Icesi, Facultad de Ciencias de la Salud, Departamento de Salud Pública, Cali, Colombia; ^4^ Fundación Valle del Lili, Centro de Investigaciones Clínicas (CIC), Cali, Colombia; ^5^ Universidad Tecnológica de Pereira, Facultad de Ciencias de la Salud, Departamento de Pediatría, Pereira, Colombia; ^6^ Fundación Valle del Lili, Unidad de Atención de Cáncer Infantil, Unidad Materno Infantil, Cali, Colombia; ^7^ Division of Critical Care and Pulmonary Medicine, Department of Pediatrics, St Jude Children’s Research Hospital, Memphis, TN, United States

**Keywords:** pediatric, cancer, central nervous system neoplasms, tumor, pediatric intensive care unit, postoperative complications, resource-limited settings

## Abstract

**Introduction:**

Central nervous system (CNS) tumors are the second most prevalent malignant neoplasms in childhood, with surgical resection as the primary therapeutic approach. The immediate postoperative period following CNS tumor resection requires intensive care to mitigate complications associated with high morbidity and mortality.

**Objective:**

The primary aim of this study is to comprehensively describe the postoperative complications observed in pediatric patients who underwent primary CNS tumor resection and were subsequently admitted to the pediatric intensive care unit (PICU) at Hospital Universitario Fundación Valle del Lili in Colombia.

**Methods:**

We conducted a cross-sectional observational analysis of pediatric patients who underwent surgery for CNS tumors and were admitted to our PICU from January 2011 to December 2021. Clinical, histopathologic, and postoperative complication data were collected. A descriptive statistical analysis was performed using measures of dispersion and central tendency with a 95% confidence interval.

**Results:**

A total of 114 patients were included, of whom 55.3% were male. The median PICU stay was 4 days (2–7). The most common tumor type was embryonal (25.4%), followed by low-grade glioma (20.1%) and high-grade glioma (14.9%). Mechanical ventilation was required in 24.5% of patients, with a median extubation time of 3 days (2–9). In the immediate postoperative period, 6.14% of patients experienced CNS hemorrhage and 3.5% experienced intracranial hypertension. Common complications included motor deficits, facial paralysis, and sensory deficits. The mortality rate was 3.5%.

**Conclusion:**

This study describes the postoperative complications, clinical challenges, and interventions observed in pediatric patients after CNS tumor resection in a resource-limited country. Our findings emphasize the importance of tailored interventions and multidisciplinary collaboration to optimize clinical outcomes. Future data comparison from centers sharing similar characteristics will play a crucial role in identifying best practices and enhancing outcomes globally.

## 1 Introduction

Pediatric central nervous system (CNS) tumors are the most common solid tumors in childhood and the leading cause of cancer-related death, accounting for 15%–20% of deaths among children aged 0 to 14 years globally (1–4). According to the Central Brain Tumor Registry of the United States, the incidence of primary CNS tumors is 6.14 cases per 100,000 children and adolescents (1, 5, 6), with 58% of these cases classified as malignant.

In high-income countries, the 5-year survival for pediatric patients with cancer exceeds 80%, reflecting the impact of early diagnosis and access to specialized healthcare services (7). However, global disparities exist, including in Latin America where the prevalence of childhood cancer is 11%, which is lower than that of Asian and African countries (8). In Colombia, CNS cancer in children is a significant health burden. The reported prevalence is 22.1% and it is the second leading cause of cancer-related death, with 47.4% of cases involving solid tumors (1, 2, 7). Moreover, 7,769 cases of childhood cancer were reported between January 2020 and January 2021, including 876 new cases. In southwestern Colombia, where our institution is geographically located, the prevalence of childhood cancer is 575 (95% CI, 545–606) per 1,000,000 individuals under the age of 18 years (2).

Precise tumor diagnosis and staging play a crucial role in determining treatment and prognosis. The World Health Organization (WHO) classification, with its 2021 update, places a strong emphasis on molecular diagnostics to distinguish tumors affecting adults from those affecting children, as this differentiation may influence treatment strategies and disease progression (9). CNS tumors in children, which originate from the brain, spinal cord, and meninges, are predominantly of primary origin, with gliomas comprising half of these tumors in children and adolescents (10, 11).

Surgical resection is the preferred treatment for CNS tumors in children. In Colombia, surgery has been the main treatment for children with CNS tumors since 2020, with an adoption rate of 98.7%. The surgical approach is tailored to the patient’s clinical condition and imaging findings (3, 12), with a focus on tumor mass reduction, intracranial hypertension management, hydrocephalus relief, or obtaining tumor samples for accurate classification. Postoperative care in a pediatric intensive care unit (PICU) is essential for continuous monitoring and early detection of complications such as infections, seizures, endocrine disturbances, or stroke (12).

Outcome data, including postsurgical data, provide valuable insights into care, risks, and opportunities for improvement in patient care. Despite Colombia’s effort to conduct epidemiological surveillance of pediatric cancer, there is limited information on postoperative outcomes following tumor resection. This study aims to describe the clinical outcomes and most common complications during the postoperative period for pediatric patients admitted to a PICU between 2011 and 2021 in a resource-limited setting that manages complex pediatric cases in southwestern Colombia.

## 2 Materials and methods

### 2.1 Study design and population

A descriptive, cross-sectional study with retrospective data collection was conducted. The study included patients aged 1 to 18 years who underwent resection of CNS tumors at our tertiary university hospital between January 2011 and December 2021 and were subsequently admitted to the PICU during the postoperative period. Patients whose surgical resections or diagnoses were conducted outside our institution without histopathologic review performed at our hospital, as well as those who underwent solely diagnostic biopsies, were excluded from the study. Data were extracted from the patients’ medical records and tabulated using a standardized form.

### 2.2 Setting

Hospital Universitario Fundación Valle del Lili is a general hospital with a significant portion of its services (25% to 30%) dedicated to pediatric patients. It serves as a referral center for high-complexity pathology in the southwestern region of Colombia. The hospital contains a PICU with 40 beds, including 10 designated for pediatric cardiovascular surgery and 30 for general medical-surgical and oncologic conditions. On average, the PICU admits approximately 200 patients per month, with 20% to 30% experiencing critical oncologic conditions. Colombia is classified as a middle-income country according to the Global Burden of Disease Study 2019 Socio-Demographic Index (13), which is an indicator of development status that correlates with health outcomes. Hospitals in this region encounter therapeutic and resource constraints and high patient volumes, resulting in frequent resource limitations.

### 2.3 Variables

The diagnoses of eligible patients are listed in **Appendix 1**. Sociodemographic factors such as age, sex, city of residence, and type of health insurance were recorded. Clinical characteristics prior to surgery were also documented, including tumor classification based on the 2016 WHO classification, medical history, and neurologic symptoms. **Supplementary Material 1** provides the complete World Health Organization (WHO) classification system used for tumor categorization in this study. Postoperative outcomes including complications such as the need for advanced interventions (e.g., invasive mechanical ventilation or vasopressors) and mortality were also analyzed. The functional status score (FSS) was used to evaluate patients’ functionality upon admission to and discharge from the PICU. Postoperative complications were categorized based on their occurrence within 24 hours (immediate), between 24 and 48 hours (early), between 48 hours and 7 days (intermediate), and beyond 7 days during the PICU stay (late). All patients were examined by pediatric intensivists, neurosurgeons, neurologists, and critical care nurses.

### 2.4 Statistical analysis

Variables were described based on their nature and distribution. The type of distribution for quantitative variables was determined using the Shapiro-Wilk test. Quantitative variables exhibiting a normal distribution were characterized using the mean and standard deviation, whereas those with non-normal distributions were described using median and interquartile range (IQR). The incidence of complications was calculated by defining the numerator as the number of cases with complications and the denominator as the total number of patients admitted to the PICU with a history of surgical tumor resection during the study period. Qualitative variables were summarized by frequencies and percentages. A 95% confidence interval was calculated. The statistical software STATA SE v18 was used for the analysis.

### 2.5 Ethical considerations

This study was approved by the Research Ethics Committee of Hospital Universitario Fundación Valle del Lili. Informed consent was not required for this study due to its retrospective nature and the use of electronic medical records. Data confidentiality was maintained throughout the study.

## 3 Results

This study included 114 patients who were admitted to the PICU immediately after undergoing CNS tumor resection. There were 63 males (55.3%) and 51 females (44.7%), with a median age of 8 years (IQR, 4–12) at the time of the first surgery. The median time from symptom onset to diagnosis was 29.5 days (IQR, 8–121), and the median time from diagnosis to surgery was 2 days (IQR, 1–8).

Before surgery, 21.1% (n=24) of patients experienced intracranial hypertension, with 12.2% (n=14) requiring emergent ventriculostomy prior to tumor resection. Due to cerebral edema pre-surgery, 78.7% (n=89) of patients received treatment with dexamethasone, and 79.8% (n=91) continued corticosteroid therapy for up to 5 days postoperatively. Only 15.7% (n=18) of patients required corticosteroid administration beyond the initial 5 days after surgery.

Regarding surgical outcomes, all patients underwent primary surgery, with 49.1% (n=56) achieving total tumor resection and 28.1% (n=32) undergoing partial resection (less than 90% resection), as documented in the neurosurgeon’s surgical notes. Surgical outcome information was not available in the medical records of 26 patients (22.8%). Tumor localization was primarily infratentorial in 51.7% of cases (n=59). The most prevalent tumor type by histology was embryonal (25.4%, n=29), followed by low-grade glioma (20.1%, n=23). Tumors were graded according to the 2016 WHO CNS Tumor Classification (14) as outlined in **Table 1**. During surgery, 19.3% (n=22) of patients required blood transfusions. Additionally, 50% (n=57) of patients had ventriculostomy placement, either concurrently with tumor resection or in the early postoperative period.

**Table 1:** Demographics and clinical characteristics of pediatric patients admitted to the PICU following CNS tumor resection (n=114).

Characteristic	Total number (%)	95% CI
**Age, median (IQR)**	8 (4-12)	-----
Sex		
Male	63 (55.3)	46.5-65.4
Female	51 (44.7)	35.4-54.3
Health insurance		
Contributory	74 (64.9)	55.4-73.6
Subsidized	40 (35.1)	26.4-44.5
Origin		
Urban	91 (79.8)	71.3-86.7
Rural	23 (20.1)	13.2-28.7
Tumor location		
Supratentorial	51 (44.7)	35.4-54.3
Infratentorial	59 (51.7)	42.2-61.2
Spinal cord	4 (3.5)	0.96-8.74
Type of histologic tumor		
Embryonal tumor	29 (25.4)	17.7-34.4
High-grade glioma	17 (14.9)	8.93-22.7
Low-grade glioma	23 (20.1)	13.2-28.7
Craniopharyngioma	7 (6.14)	2.50-12.2
Ependymal tumor	15 (13.1)	7.55-20.7
Germinal tumor	5 (4.38)	1.43-9.93
Mesenchymal tumor	5 (4.38)	1.43-9.93
Other	13 (11.4)	6.21-18.7
Tumor grade according to WHO scale		
Grade I	28 (24.6)	16.9-33.5
Grade II	16 (14.0)	8.24-21.7
Grade III	45 (39.5)	30.4-49.1
Grade IV	17 (14.9)	8.93-22.7
No information	8 (7.01)	3.07-13.3
Lansky Score at admission, median (IQR)	60 (50-80)	------
Pathological history		
History of respiratory pathology	8 (7.01)	3.07-13.3
Oncologic-hematologic	5 (4.38)	1.44-9.93
History of non-hepatic gastrointestinal pathology	1 (0.87)	0.02-4.79
Congenital heart disease	2 (1.75)	0.21-6.19
Other neurologic disorders	13 (11.4)	6.21-18.7
Type of management at diagnosis		
Surgical	107 (93.8)	87.7-97.5
Medical and surgical	7 (6.14)	2.50-12.2
Days between diagnosis and management	2 (1-8)	------
Duration of PICU stay*, median (IQR)	4 (2-7)	------
Duration of hospital stay*, median (IQR)	18 (10-29)	------
Mortality status		
Alive	110 (96.5)	91.2-99.0
Deceased	4 (3.5)	0.96-8.74
FSS prior to PICU admission, median (IQR)	6 (6-7)	------
Preoperative clinical characteristics		
Intracranial hypertension	24 (21.1)	13.9-29.6
Need for ventriculostomy	14 (12.2)	6.87-19.7
Use of dexamethasone	89 (78.7)	69.4-85.3

*Measured in days.

CI, confidence interval; CNS, central nervous system; FSS, functional status score; IQR, interquartile range; PICU, pediatric intensive care unit; WHO, World Health Organization.

After surgery, 33 patients (28.9%) had an uneventful postoperative phase. In some cases, multiple complications occurred in a single patient. Regarding the therapeutic interventions performed during the immediate postoperative phase (<24 hours), 32.5% (n=37) of patients required anticonvulsants, 17.5% (n=20) received antibiotics, 7.89% (n=9) were administered vasopressors, and 6.24% (n=7) received hyperosmolar therapy. Throughout their PICU stay, 24.5% (n=28) of patients required invasive mechanical ventilation (IMV) with a median duration of 3 days (IQR, 2–9). Additional details are shown in **Table 2**.

**Table 2:** Clinical conditions and interventions for pediatric patients admitted to the PICU following CNS tumor resection (n=114).

Intervention	Total number (%)	95% CI
Surgical outcome		
Complete resection	56 (49.1)	39.6-58.6
Partial resection	32 (28.1)	20.1-36.3
Other surgical approach (unspecified)	26 (22.8)	14.7-39.6
Requirement for intraoperative transfusion of blood products	22 (19.3)	12.5-27.7
Minutes of anesthesia, median (IQR)	254 (200-310)	------
Post-op ventriculostomy	57 (50)	40.5-59.5
Use of dexamethasone post-op < 5 days	91 (79.8)	71.3-86.7
Use of dexamethasone post-op > 5 days	18 (15.7)	9.63-23.8
Requirement for vasoactive drugs in the first 24h	9 (7.89)	3.67-14.5
Requirement for anticonvulsants in the first 24h	37 (32.5)	23.9-41.8
Antibiotic therapy in the first 24h	20 (17.5)	11.1-25.8
Type of antibiotic used		
Cephalosporins	19 (16.6)	10.3-24.7
Sulfonamides	1 (0.87)	0.02-4.79
Glycopeptides	2 (1.75)	0.21-6.19
Penicillin	3 (2.63)	0.54-7.49
Indication for antibiotics (n=20)		
Prophylactic	16 (80)	56.3-94.3
Signs and symptoms of infection	4 (20)	5.73-43.6
IMV during PICU stay	28 (24.5)	16.9-33.5
Duration of IMV*, median (IQR)	3 (2-9)	------
Gastrostomy	15 (13.2)	7.55-20.7
Tracheostomy	6 (5.26)	1.95-11.1
Hyperosmolar therapy	7 (6.14)	2.50-12.2
Fluid overload >8% on the third post-op day**	4 (3.45)	0.96-8.74

*Measured in days.

**Fluid overload = (Administered fluids minus eliminated fluids) divided by weight, multiplied by 100.

CI, confidence interval; CNS, central nervous system; IMV, invasive mechanical ventilation; IQR, interquartile range; PICU, pediatric intensive care unit; Post-op, postoperative.

**Table 3** outlines the clinical characteristics and complications during early and late postoperative periods, grouped according to their timing. In the initial 24 hours following surgery, complications included surgical bed hemorrhage (6.14%, n=7), intracranial hypertension (3.50%, n=4), and septic shock (0.87%, n=1). Regarding neurologic status at 24 hours postoperatively, 89 (78%) patients had a Glasgow Coma Scale (GCS) score between 14 and 15, 10 (8.8%) had a score between 9 and 13, and 3 (2.6%) scored below 9. In 12 patients (10.5%), the GCS score was not available due to sedation and IMV at that time. Within the first 48 hours, the most common complications were motor deficits (36.8% of patients, n=42), facial paralysis (12.2%, n=14), and sensory deficits (11.4%, n=13). Diabetes insipidus, cerebellar syndrome, seizures, and hypothalamic insufficiency occurred in fewer patients. Between 2 and 7 days postoperatively, motor deficits persisted in 30.7% of patients (n=35), with notable occurrences of facial paralysis and sensory deficits. CNS infection was reported in 1 patient (0.87%). After 7 days, the incidence of motor deficits decreased to 13.2% (n=15), but sensory deficits, facial paralysis, seizures, and endocrine disorders remained. During this period, 3 patients (2.63%) developed infectious complications.

**Table 3:** Postoperative clinical characteristics of pediatric patients admitted to the PICU following CNS tumor resection (n=114).

Clinical characteristic	Total number (%)	95% CI
Clinical characteristics within the first 24h postoperatively		
CNS hemorrhage	7 (6.14)	2.50-12.2
Intracranial hypertension	4 (3.50)	0.96-8.74
Septic shock	1 (0.87)	0.02-4.79
GCS within the first 24h		
14-15 points	89 (78%)	69.3-85.2
9-13 points	10 (8.8%)	4.28-15.5
Less than 9 points	3 (2.6%)	0.54-7.49
Not assessable	12 (10.5%)	5.55-17.6
Changes within 48h postoperatively		
Motor deficit	42 (36.8)	28.0-46.4
Facial paralysis	14 (12.2)	6.87-19.7
Sensory deficit	13 (11.4)	6.21-18.7
Diabetes insipidus	4 (3.50)	0.96-8.74
Cerebellar syndrome	3 (2.63)	0.54-7.49
Seizures	3 (2.63)	0.54-7.49
Hypothalamic insufficiency	3 (2.63)	0.54-7.49
Changes between 2 and 7 days postoperatively		
Motor deficit	35 (30.7)	22.4-40.0
Facial paralysis	11 (9.64)	4.91-16.6
Sensory deficit	10 (8.77)	4.28-15.5
Diabetes insipidus	6 (5.26)	1.95-11.1
Cerebellar syndrome	4 (3.50)	0.96-8.74
Hypothalamic insufficiency	3 (2.63)	0.54-7.49
Seizures	2 (1.75)	0.21-6.19
CNS infection	1 (0.87)	0.02-4.79
Changes after 7 days postoperatively		
Motor deficit	15 (13.2)	7.55-20.7
Sensory deficit	5 (4.38)	1.14-9.93
Facial paralysis	4 (3.50)	0.96-8.74
Seizures	4 (3.50)	0.96-8.74
Hypothalamic insufficiency	2 (1.75)	0.21-6.19
Cerebellar syndrome	2 (1.75)	0.21-6.19
Diabetes insipidus	2 (1.75)	0.21-6.19
CNS infection	2 (1.75)	0.21-6.19
Bacteremia	1 (0.87)	0.02-4.79
Mortality Status		
Alive	110 (96.5)	91.3-99.0
Deceased	4 (3.50)	0.96-8.74
Change in FSS score from admission to discharge		
1 point	15 (13.2)	7.55-20.7
2 points	8 (7.01)	3.01-13.3
More than 2 points	12 (10.5)	5.55-17.7

CNS, central nervous system; FSS, functional status score; GCS, Glasgow Coma Scale; PICU, pediatric intensive care unit.

During the study period, 3.5% of patients died (n=4) during PICU stay. Although the causes of death were multifactorial, they were primarily related to complications that developed during the postoperative period rather than to the underlying disease. The majority of these deaths were due to CNS infections, sepsis, respiratory failure, and brain death. The FSS changed in some patients between admission and discharge, with 13.2% (n=15) of patients exhibiting a 1-point increase, 7.01% (n=8) showing a 2-point increase, and 10.5% (n=12) experiencing an increase of more than 2 points. Additionally, 5.26% (n=6) required tracheostomy and 13.2% (n=15) required gastrostomy during the PICU stay due to functional impairment and residual morbidity resulting from the primary disease and/or the surgical intervention. Patients received adjuvant chemotherapy and/or radiotherapy as part of their oncologic treatment after discharge from the PICU, but data on these additional treatments were not collected.

## 4 Discussion

Postoperative complications in children after CNS tumor resection are critical and require intensive monitoring and continuous follow-up as many factors contribute to recovery and prognosis, including the patient’s preoperative condition and the type of surgery (15, 16). To date, limited data are available on the postsurgical outcomes following CNS tumor resections in Colombia. Our study reports the surgical morbidity and mortality of pediatric patients treated in a PICU of a tertiary referral center in Cali, Colombia.

The distribution of age and sex of our patients aligned with previous research conducted by Ramírez et al. and with the VIGICANCER report published in 2024 for this patient population in Colombia (17), as well as with data from other countries (1, 2, 5, 6, 11). Similarly, the anatomical location and WHO histopathologic classification were consistent with previous reports indicating that among gliomas, astrocytomas, medulloblastomas, and ependymomas are the most prevalent histologic subtypes (6, 9–11). The median PICU stay was 4 days (IQR, 2–7), which was comparable to that of other conditions managed in the PICU and did not involve prolonged stays (18, 19).

Immediate postoperative complications included surgical site hemorrhage (6.14%), intracranial hypertension (3.5%), and septic shock (0.87%). Hemodynamic complications were infrequent, with 7.89% of patients requiring vasopressor support and 6.14% receiving hyperosmolar therapy. Notably, no patients required reoperation, indicating effective clinical management and monitoring by a multidisciplinary team of pediatric intensivists, neurosurgeons, anesthesiologists, and nurses specialized in the care of critically ill children.

Neurologic assessment at 24 hours postoperatively revealed that 78% of patients were alert and oriented (GCS >14). Only a small proportion of patients (13%) had a GCS score below 9 at 24 hours, likely due to orotracheal intubation and sedation in the early postoperative period. Despite an average anesthesia duration of 4 hours, 24.5% of patients required IMV with a median duration of 3 days, which is considered a relatively short period of ventilatory support. This need was likely due to the complexity of the procedure rather than to pulmonary complications. Of these patients, 5.2% required tracheostomy placement prior to their PICU discharge.

In our study, we found that neurologic and endocrinologic complications were the most prominent complications, consistent with the existing literature (20). Specifically, motor deficits affected 36.8% of patients within 48 hours postoperatively, followed by facial paralysis (12.2%), sensory deficits (11.4%), and cerebellar syndrome (2.63%). These findings resemble those reported by Zhang et al., who documented a motor deficit prevalence of 56.8% in pediatric patients admitted to the PICU following tumor resection (21). A small proportion of our patients experienced seizures following surgery: 2.63% within the first 48 hours and 3.5% in the late postoperative period. These findings are also consistent with the existing literature, in which the reported prevalence ranges from 1.1% to 10.5% and often correlates with substantial bleeding or electrolyte imbalances, resulting in prolonged ICU stays (20, 22–24). The prophylactic use of anticonvulsants likely contributed to the reduced frequency of this complication in our pediatric cohort.

Endocrine disorders included diabetes insipidus in 5.26% of patients and hypothalamic insufficiency in 2.63% of patients within 48 hours post-surgery. The lower prevalence of these endocrine disorders in our study compared to the reported 37.1% in other studies (25) could be attributed to the limited evaluation period during the PICU stay, which may have excluded delayed-onset conditions. Other studies have linked endocrine disorders with prolonged PICU stay and increased risk of neurologic morbidity (16, 22, 26).

Postoperative infections were relatively rare in our study. One patient developed a CNS infection (0.87%) on the fifth day post-surgery, while 2 patients experienced CNS infection (1.75%) and 1 had bacteremia (0.87%) with pneumonia and surgical site infection in the late postoperative period (>7 days). These findings are consistent with publications reporting a 2.5% prevalence of CNS infection in neurosurgical patients (15, 24). Despite only 17.5% of our patients receiving immediate postoperative antibiotics, 20% displayed clear signs of infection. Although guidelines caution against postoperative antibiotic use as they do not decrease the frequency of infections (27), our findings support the use of prophylactic antibiotics to reduce the risk of surgical site infections and meningitis associated with ventriculostomy, emergent craniotomies, and early reinterventions in specific contexts (16, 22, 28).

Based on the FSS as a measurement of long-term sequelae, 10.5% of patients had residual functional morbidity after discharge, defined as more than a 2-point difference between PICU admission and discharge scores (29). Additionally, 5.26% of patients required tracheostomy and 13.2% required gastrostomy before discharge. These advanced interventions were conducted in adherence to clinical practice guidelines and adapted to each patient’s specific clinical complexity (30). Despite the low overall long-term morbidity, we must not underestimate the impact of morbidity on the quality of life and survival of pediatric patients. This is supported by findings from previous studies and further evidenced by the need for gastrostomy and tracheostomy due to severe neurologic impairment in this specific patient population (31). Therefore, proactive consideration of early tracheostomy and gastrostomy placement is crucial to facilitate timely rehabilitation and improve overall outcomes.

The mortality rate for our patient cohort was 3.5% and was attributed to various causes, including CNS infections, sepsis, ventilatory failure, and brain death, consistent with expected complications in this vulnerable population. Mortality in high-income countries has shown a decreasing trend over recent decades due to advancements in therapeutic technologies within ICUs (24, 32, 33). However, in low- and middle-income countries like Colombia, mortality remains relatively unchanged (34). Furthermore, the observed mortality in our study was higher than that from other international studies but consistent with data from countries with similar epidemiologic profiles, highlighting challenges related to healthcare access and economic constraints faced by our population.

### 4.1 Study limitations

A primary limitation of this study is its cross-sectional design involving analysis at a single time point, which limits our ability to establish causality between the studied variables. To mitigate potential information biases, rigorous quality controls were enforced during data collection and analysis. Although the sample size was adequate for this study’s objectives, future research could benefit from larger sample sizes to enhance the generalizability of the findings. These limitations emphasize the need for additional studies that can provide further insights and relevance to comparable clinical settings, especially in hospitals with resource limitations.

### 4.2 Conclusions

This study highlights the most common postoperative complications observed in pediatric patients after resection of CNS tumors and admission to the PICU. Although relatively rare, CNS hemorrhage was an immediate complication within the initial 24 hours, whereas motor deficits were the most common complications after 48 hours. Long-term sequelae included residual morbidity in 10.5% of patients and a mortality rate of 3.5%. These findings underscore the critical importance of vigilant monitoring of pediatric patients with CNS tumors post-resection. Early detection and prompt interventions are essential in mitigating residual morbidity and mortality risks. Furthermore, these data will facilitate future comparison across institutions to improve local and global outcomes.

## Data Availability

The original contributions presented in the study are included in the article/**Supplementary Material**. Further inquiries can be directed to the corresponding author.

## Appendix 1. Tumor diagnoses

### ICD-10 Codes:

- C700 Malignant neoplasm of cerebral meninges.
- C701 Malignant neoplasm of spinal meninges.
- C709 Malignant neoplasm of meninges, unspecified.
- C710 Malignant neoplasm of cerebrum, except lobes and ventricles.
- C711 Malignant neoplasm of frontal lobe.
- C712 Malignant neoplasm of temporal lobe.
- C713 Malignant neoplasm of parietal lobe.
- C714 Malignant neoplasm of occipital lobe.
- C715 Malignant neoplasm of cerebral ventricle.
- C716 Malignant neoplasm of cerebellum.
- C717 Malignant neoplasm of brainstem.
- C718 Malignant neoplasm of overlapping sites of brain.
- C719 Malignant neoplasm of brain, unspecified part.
- C720 Malignant neoplasm of spinal cord.
- C721 Malignant neoplasm of cauda equina.
- C722 Malignant neoplasm of olfactory nerve.
- C723 Malignant neoplasm of unspecified optic nerve.
- C724 Malignant neoplasm of unspecified acoustic nerve.
- C725 Malignant neoplasm of other and unspecified cranial nerves.
- C728 Lesion of contiguous sites of the brain and other parts of the central nervous system.
- C729 Malignant neoplasm of the central nervous system, unspecified.
- D320 Benign neoplasm of cerebral meninges.
- D321 Benign neoplasm of spinal meninges.
- D329 Benign neoplasm of meninges, unspecified.
- D330 Benign neoplasm of brain, supratentorial.
- D331 Benign neoplasm of brain, infratentorial.
- D332 Benign neoplasm of brain, unspecified.
- D333 Benign neoplasm of cranial nerves.
- D334 Benign neoplasm of spinal cord.
- D337 Benign neoplasm of other specified parts of the central nervous system.
- D339 Benign neoplasm of central nervous system, unspecified.

## References

[1] Cohen AR. Brain tumors in children. N Engl J Med. (2022) 386:1922–31. doi: 10.1056/NEJMra2116344 35584157

[2] Costo C deA. Situación del cáncer en la población pediátrica atendida en el SGSSS de Colombia 2020. In: CUENTA DE ALTO COSTO Fondo Colombiano de Enfermedades de Alto Costo, Bogota D. C. (2021).

[3] Hanson DR Atlas MP. Central nervous system Malignancies. Lanzkowskys Man Pediatr Hematol Oncol. (2016), 453–72. doi: 10.1016/B978-0-12-801368-7.00023-5

[4] Alegría-Loyola MA Galnares-Olalde JA Mercado M. Artículos de revisión Tumores del sistema nervioso central. Rev Med Inst Mex Seguro Soc. (2017) 55:330–70. 28440987

[5] Ostrom QT Cioffi G Waite K Kruchko C Barnholtz-Sloan JS. CBTRUS statistical report: primary brain and other central nervous system tumors diagnosed in the United States in 2014-2018. Neuro-Oncol. (2021) 23:III1–105. doi: 10.1093/neuonc/noab200 PMC8491279 34608945

[6] Nieves-Cuervo GM Manrique-Hernández EF Ojeda-Rincón SA Galvis-Pabón S. Tumores del sistema nervioso central en población pediátrica entre 0 y 14 años. UNAB. (2016) 19(2):124–33. doi: 10.29375/01237047.2191

[7] Ortega FV García AA Pérez TM. Tumores cerebrales en niños. Pediatría Integral. (2016) 10:401–11.

[8] Laso EL González MEM. Tumores cerebrales infantiles, semiología neurológica y diagnóstico. Asocación Esp Pediatría. (2022), 151–8.

[9] Louis DN Perry A Wesseling P Brat DJ Cree IA Figarella-Branger D. The 2021 WHO classification of tumors of the central nervous system: A summary. Neuro-Oncol. (2021) 23:1231–51. doi: 10.1093/neuonc/noab106 PMC8328013 34185076

[10] Toro-Moreno AC Serna-Velez L Gallego-González D Jaramillo-Jaramillo LI Martínez-Sánchez LM Álvarez-Hernández LF. Central nervous system tumors in pediatrics: Present and future of diagnostic approach, Tumores de sistema nervioso central en pediatría: Presente y futuro del abordaje diagnóstico. Revista Ecuatoriana de Neurologia. (2017) 26.

[11] Gómez FV Ortega EC Atienza ÁL. Tumores cerebrales en niños. Pediatría Integral. (2021) 15:357–66.

[12] Linabery AM Ross JA. Trends in childhood cancer incidence in the U.S. (1992-2004). Cancer. (2008) 112:416–32. doi: 10.1002/cncr.v112:2 18074355

[13] Global burden of Disease Study 2019 (GBD 2019) Socio-Demographic Index (SDI) 1950–2019. United States: GHDx. (2024). Available at: https://ghdx.healthdata.org/record/ihme-data/gbd-2019-socio-demographic-index-sdi-1950-2019.

[14] Louis DN Perry A Reifenberger G von Deimling A Figarella-Branger D Cavenee WK. The 2016 world health organization classification of tumors of the central nervous system: a summary. Acta Neuropathologica. (2016) 131:803–20. doi: 10.1007/s00401-016-1545-1 27157931

[15] Wach J Banat M Borger V Vatter H Haberl H Sarikaya-Seiwert S. Intraoperative MRI-guided resection in pediatric brain tumor surgery: A meta-analysis of extent of resection and safety outcomes. J Neurological Surgery Part A: Cent Eur Neurosurg. (2021) 82:64–74. doi: 10.1055/s-0040-1714413 32968998

[16] Fernández de Sevilla Estrach M Lasaosa FJC Matute SS Quesada AG Rico AP. Postoperatorio de tumores cerebrales en la unidad de cuidados intensivos pediátricos. Pediatr marzo. (2009) 70:282–6. doi: 10.1016/j.anpedi.2008.10.015 19409246

[17] Ramirez O Piedrahita V Ardila J Pardo C Cabrera-Bernal E Lopera J. Primary central nervous system tumors survival in children in ten Colombian cities: a VIGICANCER report. Front Oncol. (2024) 13. doi: 10.3389/fonc.2023.1326788 PMC10949889 38505512

[18] Kirk AHP Sng QW Zhang LQ Wong JJM Puthucheary J Lee JH. Characteristics and outcomes of long-stay patients in the pediatric intensive care unit. J Pediatr Intensive Care. (2018) 7(1):1–6. doi: 10.1055/s-0037-1601337 PMC6260322 31073460

[19] Shrestha AK Bhattarai S Paudel P Basel PL. Paediatric intensive care unit of tertiary level hospital of Nepal. J Nepal Paediatr Soc. (2020) 40(1):28–33. doi: 10.3126/jnps.v40i1.28853

[20] Sangtongjaraskul S Lerdsirisopon S Sae-Phua V Kanta S Kongkiattikul L. Factors influencing prolonged intensive care unit length of stay after craniotomy for intracranial tumor in children: A 10-year analysis from A university hospital. Indian J Crit Care Med. (2023) 27:205–11. doi: 10.5005/jp-journals-10071-24418 PMC10028711 36960121

[21] Zhang Z Wu Y Zhao X Ji W Li L Zhai X. Neurosurgical short-term outcomes for pediatric medulloblastoma patients and molecular correlations: a 10-year single-center observation cohort study. Neurosurg Rev. (2024) 47:283. doi: 10.1007/s10143-024-02526-6 38904885

[22] Mishra N Rath GP Rajagopalan V Doddamani R Chaturvedi A. Perioperative management of pediatric brain tumors: A retrospective analysis. Neurol India. (2022) 70:1095–101. doi: 10.4103/0028-3886.349578 35864645

[23] Spentzas T Escue JE Patters AB Varelas PN. Brain tumor resection in children: Neurointensive care unit course and resource utilization. Pediatr Crit Care Med. (2010) 11:718–22. doi: 10.1097/PCC.0b013e3181d907fa 20308930

[24] Lassen B Helseth E Egge A Due-Tønnessen BJ Rønning P Meling TR. Surgical mortality and selected complications in 273 consecutive craniotomies for intracranial tumors in pediatric patients. Neurosurgery. abril. (2012) 70:936–43. doi: 10.1227/NEU.0b013e31823bcc61 21993188

[25] Heo J Lee HS Hwang JS Noh OK Kim L Park JE. Prevalence of endocrine disorders in childhood brain tumor survivors in South Korea. In Vivo. (2019) 33:2287–91. doi: 10.21873/invivo.11735 PMC6899123 31662569

[26] Manzanares W Aramendi I Langlois PL Biestro A. Hiponatremias en el paciente neurocrítico: Enfoque terapéutico basado en la evidencia actual. Medicina Intensiva. (2015) 39:234–43. doi: 10.1016/j.medin.2014.11.004 25593019

[27] de Jonge SW Boldingh QJJ Solomkin JS Dellinger EP Egger M Salanti G. Effect of postoperative continuation of antibiotic prophylaxis on the incidence of surgical site infection: a systematic review and meta-analysis. Lancet Infect Dis. (2020) 20:1182–92. doi: 10.1016/S1473-3099(20)30084-0 32470329

[28] Korinek AM Baugnon T Golmard JL Effenterre RV Coriat P Puybasset L. Risk factors for adult nosocomial meningitis after craniotomy: Role of antibiotic prophylaxis. Neurosurgery. (2006) 59:126–32. doi: 10.1227/01.neu.0000243291.61566.21 16823308

[29] Pollack MM Holubkov R Glass P Dean JM Meert KL Zimmerman J. The functional status score (FSS): A new pediatric outcome measure. Pediatrics. (2009) 124:e18. doi: 10.1542/peds.2008-1987 19564265 PMC3191069

[30] Ramos-Clason EC Tuñón-Pitalua MC Rivas-Muñoz FA Veloza-Cabrera LA. Tumores primarios del sistema nervioso central en Cartagena, 2001– 2006. Rev salud pública [Internet]. (2010) 12:257–6. 10.1590/s0124-00642010000200009 21031236

[31] Masoudi MS Taheri R Zoghi S. Predictive factors for postoperative tracheostomy requirement in children undergoing surgical resection of medulloblastoma. World Neurosurg. (2021) 150:e746–9. doi: 10.1016/j.wneu.2021.03.129 33812068

[32] Chalif EJ Morshed RA Oh T Ore CD Aghi MK Gupta N. Neurosurgical outcomes for pediatric central nervous system tumors in the United States. Neurosurgery. (2024) 92:407–20. doi: 10.1227/neu.0000000000002215 36637275

[33] Smith ER Butler WE Barker FG Pollack IF Sutton LN Ellenbogen RG. Craniotomy for resection of pediatric brain tumors in the United States, 1988 to 2000: effects of provider caseloads and progressive centralization and specialization of care. Neurosurgery. (2004) 54:553–65. doi: 10.1227/01.NEU.0000108421.69822.67 15028128

[34] Riaño I Bravo P Bravo LE García LS Collazsos P Carrascal E. Incidence, mortality, and survival trends of primary CNS tumors in cali, Colombia, from 1962 to 2019. JCO Glob Oncol. (2020) 6:1712–20. doi: 10.1200/GO.20.00368 PMC7713581 33156716

