# Supplementary material for: Postoperative complications after central nervous system tumor resection in pediatric patients admitted to an intensive care unit in Colombia
Source: Front Oncol. 2024 Dec 6;14:1491943. doi: 10.3389/fonc.2024.1491943 (PMC11659208; doi:10.3389/fonc.2024.1491943)
Supplement: Supplementary file 2 [file Table1.docx]

**The 2016 World Health Organization Classification of Tumors of the Central Nervous System**

1. **Diffuse astrocytic and oligodendroglial tumours**

| **Tumor Type** | **ICD-O Code** |
| --- | --- |
| Diffuse astrocytoma, IDH-mutant | 9400/3 |
| Gemistocytic astrocytoma, IDH-mutant | 9411/3 |
| Diffuse astrocytoma, IDH-wildtype | 9400/3 |
| Diffuse astrocytoma, NOS | 9400/3 |
| Anaplastic astrocytoma, IDH-mutant | 9401/3 |
| Anaplastic astrocytoma, IDH-wildtype | 9401/3 |
| Anaplastic astrocytoma, NOS | 9401/3 |
| Glioblastoma, IDH-wildtype | 9440/3 |
| Glioblastoma, NOS | 9440/3 |
| Giant cell glioblastoma | 9441/3 |
| Gliosarcoma | 9442/3 |
| Epithelioid glioblastoma | 9440/3 |
| Glioblastoma, IDH-mutant | 9440/3 |
| Diffuse midline glioma, H3 K27M-mutant | 9385/3 |
| Oligodendroglioma, IDH-mutant and 1p/19q-codeleted | 9450/3 |
| Oligodendroglioma, NOS | 9450/3 |
| Anaplastic oligodendroglioma, IDH-mutant and 1p/19q-codeleted | 9451/3 |
| Anaplastic oligodendroglioma, NOS | 9451/3 |

1. **Other astrocytic tumours**

| **Tumor Type** | **ICD-O Code** |
| --- | --- |
| Pilocytic astrocytoma | 9421/1 |
| Pilomyxoid astrocytoma | 9425/3 |
| Subependymal giant cell astrocytoma | 9384/1 |
| Pleomorphic xanthoastrocytoma | 9424/3 |
| Anaplastic pleomorphic xanthoastrocytoma | 9424/3 |

1. **Ependymal tumours**

| **Tumor Type** | **ICD-O Code** |
| --- | --- |
| Ependymoma, NOS | 9391/3 |
| Myxopapillary ependymoma | 9394/1 |
| Subependymoma | 9383/1 |
| Tanycytic ependymoma | 9391/3 |
| Clear cell ependymoma | 9391/3 |
| Papillary ependymoma | 9391/3 |
| RELA fusion-positive ependymoma | 9391/3 |

1. **Other gliomas**

| **Tumor Type** | **ICD-O Code** |
| --- | --- |
| Astroblastoma | 9430/3 |
| Chordoid glioma of the third ventricle | 9444/1 |
| Angiocentric glioma | 9431/1 |

1. **Choroid plexus tumours**

| **Tumor Type** | **ICD-O Code** |
| --- | --- |
| Choroid plexus papilloma | 9390/0 |

1. **Neuronal and mixed neuronal-glial tumours**

| **Tumor Type** | **ICD-O Code** |
| --- | --- |
| Dysembryoplastic neuroepithelial tumour | 9413/0 |
| Gangliocytoma | 9492/0 |
| Ganglioglioma | 9505/1 |
| Desmoplastic infantile ganglioglioma | 9505/1 |
| Dysplastic cerebellar gangliocytoma (Lhermitte-Duclos disease) | 9493/0 |
| Papillary glioneuronal tumour | 9509/1 |
| Rosette-forming glioneuronal tumour | 9509/1 |
| Central neurocytoma | 9506/1 |
| Extraventricular neurocytoma | 9506/1 |
| Paraganglioma | 8680/1 |

1. **Tumours of the pineal region**

| **Tumor Type** | **ICD-O Code** |
| --- | --- |
| Pineocytoma | 9361/1 |
| Pineal parenchymal tumour of intermediate differentiation | 9362/3 |
| Pineoblastoma | 9362/3 |
| Papillary tumour of the pineal region | 9393/3 |

1. **Embryonal tumours**

| **Tumor Type** | **ICD-O Code** |
| --- | --- |
| Medulloblastoma, genetically defined | 9475/3 |
| Medulloblastoma, WNT-activated | 9475/3* |
| Medulloblastoma, SHH-activated and TP53-mutant | 9475/3* |
| Medulloblastoma, SHH-activated and TP53-wildtype | 9471/3* |
| Medulloblastoma, non-WNT/non-SHH | 9475/3* |
| Medulloblastoma, histologically defined | 9470/3 |
| Medulloblastoma, NOS | 9470/3 |
| Medulloblastoma, desmoplastic/nodular | 9471/3 |
| Medulloblastoma with extensive nodularity | 9471/3 |
| Medulloblastoma, large cell/anaplastic | 9474/3 |
| Embryonal tumour with multilayered rosettes, C19MC-altered | 9478/3 |
| Embryonal tumour with multilayered rosettes, NOS | 9478/3 |
| CNS neuroblastoma, NOS | 9500/3 |
| CNS ganglioneuroblastoma | 9490/3 |
| Medulloepithelioma | 9501/3 |
| Ependymoblastoma | 9509/3 |

1. **Tumours of the cranial and paraspinal nerves**

| **Tumor Type** | **ICD-O Code** |
| --- | --- |
| Schwannoma | 9560/0 |

1. **Melanotic schwannoma**

| **Tumor Type** | **ICD-O Code** |
| --- | --- |
| Melanotic schwannoma | 9560/1 |
| Neurofibroma | 9540/0 |
| Atypical neurofibroma / Plexiform neurofibroma | 9550/0 |
| Perineurioma | 9571/0 |
| Malignant peripheral nerve sheath tumour (MPNST) | 9540/3 |
| Epithelioid MPNST | 9540/3 |
| MPNST with perineurial differentiation | 9540/3 |

1. **Meningiomas**

| **Tumor Type** | **ICD-O Code** |
| --- | --- |
| Meningioma | 9530/0 |
| Meningothelial meningioma | 9530/0 |
| Fibrous meningioma | 9530/0 |
| Transitional meningioma | 9530/0 |
| Psammomatous meningioma | 9530/0 |
| Angiomatous meningioma | 9530/0 |
| Microcystic meningioma | 9530/0 |
| Secretory meningioma | 9530/0 |
| Lymphoplasmacyte-rich meningioma | 9530/0 |
| Metaplastic meningioma | 9530/0 |
| Chordoid meningioma | 9530/1 |
| Clear cell meningioma | 9538/1 |
| Atypical meningioma | 9539/1 |
| Rhabdoid meningioma | 9538/1 |
| Papillary meningioma | 9538/1 |
| Anaplastic (malignant) meningioma | 9530/3 |

1. **Mesenchymal, non-meningothelial tumours**

| **Tumor Type** | **ICD-O Code** |
| --- | --- |
| Solitary fibrous tumour / haemangiopericytoma | 8815/0 |
| Grade 1 | 8815/0 |
| Grade 2 | 8815/1 |
| Grade 3 | 8815/3 |
| Haemangioblastoma | 9161/1 |
| Meningeal haemangiopericytoma | 9133/0 |
| Meningeal haemangioendothelioma | 9120/3 |
| Alveolar soft part sarcoma | 9581/3 |
| Angiosarcoma | 9120/3 |
| Ewing sarcoma / PNET | 9260/3 |
| Leiomyosarcoma | 8890/3 |
| Lipoma | 8850/0 |
| Liposarcoma | 8850/3 |
| Myxopapillary fibrosarcoma | 8811/1 |
| Myxofibrosarcoma | 8811/3 |
| Benign fibrous histiocytoma | 8830/0 |
| Undifferentiated pleomorphic sarcoma / malignant fibrous histiocytoma | 8802/3 |
| Osteoma | 9180/0 |
| Osteochondroma | 9210/0 |
| Osteosarcoma | 9180/3 |

1. **Melanocytic tumours**

| **Tumor Type** | **ICD-O Code** |
| --- | --- |
| Melanocytoma | 8728/0 |
| Meningeal melanocytoma | 8728/0 |
| Meningeal melanoma | 8720/3 |
| Meningeal melanomatosis | 8720/3 |

1. **Lymphomas**

| **Tumor Type** | **ICD-O Code** |
| --- | --- |
| Diffuse large B-cell lymphoma of the CNS | 9680/3 |
| Immunodeficiency-associated CNS lymphomas | 9680/3 |
| AIDS-related diffuse large B-cell lymphoma | 9680/3 |
| EBV-positive diffuse large B-cell lymphoma, NOS | 9680/3 |
| Lymphomatoid granulomatosis | 9761/3 |
| Primary Burkitt lymphoma | 9732/3 |
| Low-grade B-cell lymphomas of the CNS | 9680/3 |
| T-cell and NK/T-cell lymphomas of the CNS | 9680/3 |
| Anaplastic large cell lymphoma, ALK-positive | 9714/3 |
| Anaplastic large cell lymphoma, ALK-negative | 9702/3 |
| MALT lymphoma of the CNS | 9699/3 |

1. **Histiocytic tumours**

| **Tumor Type** | **ICD-O Code** |
| --- | --- |
| Langerhans cell histiocytosis | 9751/3 |
| Erdheim-Chester disease | 9751/3 |
| Rosai-Dorfman disease | 9762/3 |
| Juvenile xanthogranuloma | 9755/3 |

1. **Germ cell tumours**

| **Tumor Type** | **ICD-O Code** |
| --- | --- |
| Germinoma | 9064/3 |
| Embryonal carcinoma | 9070/3 |
| Choriocarcinoma | 9100/3 |
| Yolk sac tumour | 9071/3 |
| Teratoma | 9080/1 |
| Immature teratoma | 9080/3 |
| Teratoma with malignant transformation | 9084/3 |
| Mixed germ cell tumour | 9085/3 |

1. **Tumours of the sellar region**

| **Tumor Type** | **ICD-O Code** |
| --- | --- |
| Craniopharyngioma | 9350/1 |
| Adamantinomatous craniopharyngioma | 9351/1 |
| Papillary craniopharyngioma | 9352/1 |
| Pituicytoma | 9432/1 |
| Granular cell tumour of the sellar region | 9440/1 |
| Spindle cell oncocytoma | 8290/1 |

1. **Metastatic tumours to the CNS**
